# Pre-existing mutations related to tenofovir in chronic hepatitis B patients with long-term nucleos(t)ide analogue drugs treatment by ultra-deep pyrosequencing

**DOI:** 10.18632/oncotarget.11840

**Published:** 2016-09-02

**Authors:** Xiaxia Zhang, Minran Li, Hongli Xi, Renwen Zhang, Jianhong Chen, Yu Zhang, Xiaoyuan Xu

**Affiliations:** ^1^ Department of Infectious Disease, Peking University First Hospital, Xicheng District, Beijing 100034, China; ^2^ Division of Liver Disease, The Fifth Hospital of Shijiazhuang, Hebei Medical University, Shijiazhuang 050023, China

**Keywords:** hepatitis B virus, resistance, multi-drugs therapy, tenofovir, ultra-deep pyrosequencing

## Abstract

**Aims:**

The dynamics of resistance-associated mutations under combination therapy were explored.

**Methods:**

A total of 46 patients were classified into adefovir (n=14) and entecavir (n=32) groups. In the adefovir (ADV) group, six patients receiving combined therapy were DNA-positive after more than 3 years of therapy. Ultra-deep pyrosequencing was used to analyze the dynamics of multi-drugs resistance mutations.

**Results:**

At baseline, all 46 treatment-naïve patients harbored rtA181V/T substitutions (1.2%-4.6%) and rtN236T substitutions (1.6%-6.1%). In the ADV group, eight patients with long-term treatment were consecutively HBV DNA-positive for more than 3 years. During treatment, the rtA181T resistance-associated site appeared with increasing frequency in six of eight patients (NOs. 1-6), and two patients (NOs.4 and 8) carrying the rtA181T resistance mutations increasingly showed high levels of rtN236T. One patient (NO. 8) experienced virological breakthrough. Other known pre-existing mutations showed no dynamic fluctuations, including in rtA194T, rtP177G, rtF249A, and rtD263E. In addition to the common substitutions, some previously unknown amino acid substitutions, such as rtD134N, rtL145M/S, rtF151Y/L, rtR153Q, and rtS223A, should be further studied.

**Conclusions:**

HBV-resistance substitutions conferring to nucleoside analogs are present at baseline. The dynamics of the HBV RT-region quasispecies variation are heterogeneous and complex.

## INTRODUCTION

Hepatitis B virus (HBV) infection remains a critical global health issue that can cause serious liver-related morbidity and mortality [1]. The latest research data show that there are approximately 350 million chronic hepatitis B (CHB) patients [2]. In the clinical context, the most widely used antiviral therapies are the approved five oral antiviral drugs, including nucleosides (lamivudine, telbivudine, and entecavir) and nucleotides (adefovir, and tenofovir), which directly inhibit the HBV reverse transcriptase enzyme to suppress viral replication. However, their long-term use is required in most patients. Due to the lack of viral-encoded RNA-dependent DNA polymerase proofreading activity and the high rate of viral replication, mutations are common and can lead to treatment failure [3]. Drug-resistant mutants are now a major problem [4]. Several nucleos(t)ide-analogue (NA) drugs, including lamivudine, telbivudine, entecavir and adefovir, can be rendered useless by resistance-associated mutations.

Some reports have demonstrated that the NAs may cause the accumulation of related resistantance-associated variants, which may supress the subsequent effect of therapy. Because of its high barrier to resistance, tenofovir disoproxil fumarate (TDF) has been recommended as a first-line anti-HBV drug, tenofovir-based therapy is also recommended as a rescue therapy in patients with nucleoside-resistant hepatitis b virus [5, 6]. It has been reported that rtA181T+ rtN236T, rtA194T, rtP177G and rtF249A may increase viral replication and reduce the susceptibility to tenofovir [7–9]. HBV mutants resistant to adefovir (ADV) monotherapy are complex and diverse, including rtA181T, rtV214A, and rtN236T [10]. However, adefovir is still widely used because of its low price. The majority of the CHB population experienced treatment with multiple NAs. Some reports have studied the dynamics of resistance mutations with short-time adefovir monotherapy [11]. However, the data on the dynamics of combination therapy with multiple long-term NAs are very limited.

Patients should be able to choose an optimal treatment program, especially if they have previously taken adefovir dipivoxil. However, the data on TDF in Asian countries, especially for patients who experienced treatment failure with long-term multiple NAs, are limited. Thus, the cost of and need for essentially life-long treatment pose considerable challenges.

## RESULTS

### Patients and samples

Finally, 46 CHB patients were included in the study: 32 patients were treated with ETV, and 14 patients were treated with ADV. The clinical characteristics of all patients at baseline are listed in Table 1. In the ADV group, eight patients (NOs.1 to 8) with long-term ADV and other antiviral drugs were consecutively tested for more than 3 years, and among them, six patients were given multi-drug therapy, one patient was given long-term ADV monotherapy, and one patient underwent virological breakthrough.

**Table 1:** Clinical characteristics of the 46 treatment-naïve chronic hepatitis B patients

	ADV group (n=14)	ETV group (n=32)
Gender(male/female)	12/2	26/6
Age(years)	36.50±10.92	34.30±11.66
HBeAg+ (%)	8(57.14%)	18(56.25%)
ALT (IU/mL)	130.5(75.5-190.3)	98.5(57-197.5)
HBV DNA (log10IU/mL)	6.37±1.56	7.71±0.77
HBsAg (log10 IU/mL)	3.60±0.65	4.11±0.78
HBV genotype		
B(%)	3(21.43%)	4(12.50%)
C(%)	9(64.28%)	16(50.00%)
Undetermined	2(14.29%)	12(37.50%)

A total of 46 patients were classified as ADV and ETV groups. At baseline, we analyze the characteristics of the two groups.

HBeAg, hepatitis B e antigen; ALT, alanine aminotransferase; HBsAg, hepatitis B surface antigen;

### NA-related resistance mutations at baseline

At baseline, the frequencies of NAr substitutions in the two groups were determined by UDPS (Table 2). All treatment-naïve patients carried rtA181V/T substitutions (ranging from 1.1% to 8.7%) and rtN236T substitutions (ranging from 1.4% to 6.1%). Other substitutions (rtI169T/V, rtP177G, rtT184A/I, rtA194V/T, rtV214A, rtQ215R/H, rtF249A, and rtM250V) were present at low levels (<1%). In the ADV group, the rtA181V/T substitutions rang from 1.2% to 8.7%, and the rtN236T substitutions rang from 1.4% to 6%. Five patients harbored rtL180M substitutions (2.1% / 12.5%), and they also presented rtM204I/V substitutions (2.9% / 7.5%) and rtS202G substitutions (2.9% / 3.3%), which are all known to confer resistance to ETV.

**Table 2:** Frequencies of amino acid substitutions in the 46 treatment-naïve chronic hepatitis B patients

Groups	Patient	HBV DNA(log	rtI169T/V	rtV173A/M	rtP177G	rtL180M	rtA181V/T	rtT184A/I	rtA194V/T	rtS202G	rtM204I/V	rtV214A	rtQ215R/H	rtN236T	rtF249A	rtM250V
	(Nos.)	10 IU/mL)	(%)	(%)	(%)	(%)	(%)	(%)	(%)	(%)	(%)	(%)	(%)	(%)	(%)	(%)
ADV	1*	8.38	<	<	<	2.4	4.7	<	<	3.3	3.4	<	<	6	<	<
	2*	4.99	<	<	<	12.5	4.7	<	<	3.2	7	<	<	5.7	<	<
	3*	7.2	<	<	<	<	1.4	<	<	1	1	<	<	1.8	<	<
	4*	7.81	<	<	<	<	1.2	<	<	<	<	1.2	<	1.4	<	<
	5*	7.8	<	<	<	<	1.2	<	<	1.1	1.1	<	<	1.8	<	<
	6*	7.4	<	1.6	<	10.5	8.7	<	<	<	7.5	<	<	1.7	<	<
	7*	8.08	<	<	<	<	1.2	<	<	<	<	1	<	1.5	<	<
	8*	7.53	<	<	<	<	1.3	<	<	<	<	<	<	1.6	<	<
	9	7.94	<	<	<	2.1	4.5	<	<	2.9	2.9	<	<	6	<	<
	10	5.75	<	<	<	<	1.4	<	<	<	<	<	<	1.6	<	<
	11	7.06	<	<	<	<	1.4	<	<	1	1	<	<	1.9	<	<
	12	6.16	<	<	<	<	2	<	<	1.1	1.2	2.7	<	2.2	<	<
	13	7.38	<	<	<	2.2	4.1	<	<	3.1	3.1	<	<	6	<	<
	14	7.68	<	<	<	<	1.4	<	<	1	1	<	<	1.7	<	<
ETV	15*	7.82	<	<	<	2.1	3.8	<	<	3.4	3.5	<	<	6.1	<	<
	16*	8.59	<	<	<	<	1.4	<	<	1	<	<	<	1.9	<	<
	17*	8.75	<	<	<	<	1.2	<	<	<	<	<	<	1.6	<	<
	18*	8.28	<	<	<	<	1.3	<	<	1	1	<	<	1.9	<	<
	19*	6.34	<	<	<	<	2.7	<	<	1	1	<	<	2.1	<	<
	20	7.85	<	<	<	<	1.4	<	<	1	1.1	<	<	2.1	<	<
	21	7.26	<	<	<	<	1.3	<	<	1	<	<	<	1.6	<	<
	22	7.31	<	<	<	2.5	4.3	<	<	3.2	3.4	<	<	5.9	<	<
	23	7.11	<	<	<	1.8	3.9	<	<	2.6	2.7	<	<	4.7	<	<
	24	8.56	<	<	<	<	1.2	<	<	<	<	<	<	1.6	<	<
	25	6.35	<	<	<	2.6	2.4	<	<	1	3	1	<	1.9	<	<
	26	8.44	<	<	<	<	1.3	<	<	1	1	<	<	1.9	<	<
	27	8.07	<	<	<	<	1.4	<	<	1.1	1.1	<	<	1.8	<	<
	28	7.19	<	<	<	<	1.7	<	<	1	<	<	<	1.6	<	<
	29	8.23	<	<	<	<	1.4	<	<	1	1	<	<	2	<	<
	30	7.98	<	<	<	<	1.4	<	<	1	1	<	<	1.8	<	<
	31	8.31	<	<	<	1.8	4.1	<	<	2.7	2.9	<	<	6.1	<	<
	32	6.31	<	<	<	<	1.5	<	<	1	1	1.1	<	1.7	<	<
	33	8.12	<	<	<	<	1.1	<	<	<	<	<	<	1.5	<	<
	34	7.34	<	<	<	<	1.6	<	<	1	1	<	<	1.8	<	<
	35	3.51	<	1.7	<	<	1.2	<	<	1	1	<	<	1.7	<	<
	36	3.67	<	<	<	2.4	4.6	<	<	3.2	3.2	<	<	6.1	<	<
	37	7.56	<	<	<	<	1.3	<	<	1	<	<	<	1.8	<	<
	38	6.98	<	<	<	<	1.4	<	<	1	1	<	<	1.8	<	<
	39	6.82	<	<	<	<	3.6	<	<	<	<	<	<	1.8	<	<
	40	8.24	<	<	<	<	2	<	<	<	1.4	<	<	1.8	<	<
	41	5.24	<	<	<	<	2.4	<	<	1.4	1.4	<	<	2.8	<	<
	42	5.93	<	<	<	<	1.5	<	<	1	1	<	<	1.9	<	<
	43	7.12	<	<	<	2.3	4.6	<	<	3.5	3.6	<	<	6.1	<	<
	44	6.12	<	<	<	<	1.2	<	<	1	1.1	<	6.7	1.8	<	<
	45	7	<	<	<	<	1.2	<	<	<	<	<	<	1.8	<	<
	46	8.2	<	<	<	<	1.6	<	<	<	<	<	<	1.7	<	<

Several reports have demonstrated that pre-existing variants may cause the accumulation of resistant variants in treatment-naïve hepatitis patients. Next-generation sequencing techniques can detect the frequency of less than 1% of previously known and novel substitutions

(*): patients do not achieved virological response after treatment for 1 or 2 years. (<) indicates the frequencies of amino acid substitutions <1% tested by ultra-deep sequencing.

**Figure 1:**
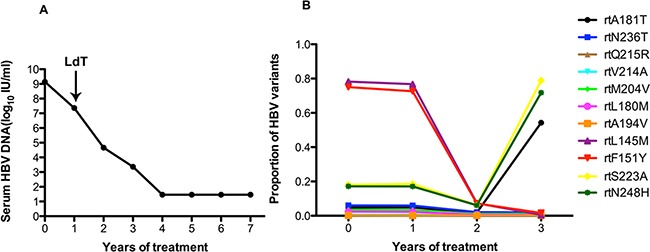
The dynamic changes in HBV DNA levels and resistance-associated variants in the RT domains during ADV+LdT treatment in patient 1 **A.** The level of serum HBV DNA declined gradually and was undetectable after 4 years of treatment and 7 years of followed-up. **B.** The dynamic changes in the RT domains of HBV variants, as derermined by UDPS. At baseline, this patient harbored the rtL180M (2.4%), rtM204I/V (3.4%), rtS202G (3.3%), rtA181V/T (4.7%), and rtN236T (6%) substitutions. The resistance-associated variants show minor fluctuations after the addition of LdT therapy in the first year. A wave of resistance-associated variants was detected at year 3, when the frequency of the rtA181T substitution rose from 1.91% to 54.23%. Meanwhile some certain non-resistance-related mutations of rtL145M (ranging from 76.77% to 7.30%) and rtF151Y (ranging from 72.61% to 7.15%) had decreased significantly in frequency with the addition of LdT. However, the frequencies of the rtS223A and rtN248H mutations declined in the first year with the addition of LdT and then increased significantly in the second year.

In patient 1, the serum DNA level declined slowly over one year of ADV treatment, and LdT was added at the end of the first year. The serum DNA level declined gradually and was undetectable after 4 years (Figure 1A). Figure 1B shows the dynamic changes in the RT domains of HBV variants, as determined by UDPS. At baseline, this patient carried with rtL180M (2.4%), rtM204I/V (3.4%), rtS202G (3.3%), rtA181V/T (4.7%), and rtN236T (6%) substitutions. The frequencies of resistantance-associated variants showed minor fluctuations after LdT was added in the first year. A wave of resistantance-associated variants was detected at 3 year, as the frequency of the rtA181T substitution rose from 1.91% to 54.23%. Meanwhile some non-resistance-related mutations, such as rtL145M (ranging from 76.77% to 7.30%) and rtF151Y (ranging from 72.61% to 7.15%) had decreased significantly with the addition of LdT. However, the frequencies of the rtS223A and rtN248H mutations declined in the first year after combined with LdT but were significantly increased at the two-year mark.

**Figure 2:**
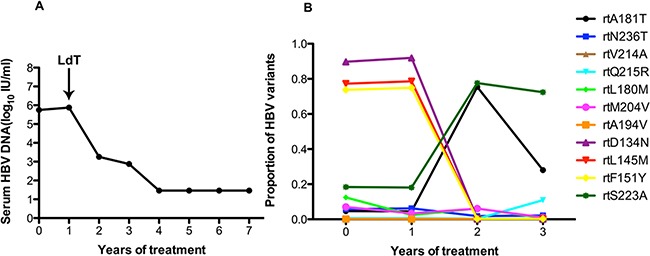
The dynamic changes in HBV DNA levels and resistance-associeted variants in the RT domains during ADV+LdT treatment in patient 2 **A.** The serum HBV DNA level was undetectable after 4 years of treatment. **B.** Patient 2 added LdT to after treatment with ADV for 1 year. At baseline, this patient harbored the rtA181T (4.7%), rtN236T (5.7%), rtL180M (12.5%), rtS202G (3.2%), and rtM204I/V (7%) substitutions. The frequencies of the resistant variants show minor fluctuations after the addition of LdT one year after beginning treatment. The frequency of the rtA181T substitution increased to 75.41% in the first year with the addition of LdT, then declined to 28.07% in the second year. In contrast, the rtQ215R substitution increased to 10.89% in the second year with multi-drugs. Some certain non-resistance mutations of rtD134N (ranging from 91.97% to 0.21%), rtL145M (ranging from 78.58% to 0.27%) and rtF151Y (ranging from 74.89% to 0.27%) had decreased significantly with the addition of LdT. However, the frequency of the rtS223A mutation increased significantly (ranging from 18.14% to 77.62%) with the addition of LdT.

As in patient 1, patient 2 also added LdT to the treatment regimen after treatment with ADV for 1 year. The serum DNA level did not change in the first year with ADV monotherapy. After this patient added LdT to the therapeutic regimen at the end of the first year, the serum DNA level declined gradually and was undetectable after 4 years (Figure 2A). At baseline, patient 2 harbored the rtA181T (4.7%), rtN236T (5.7%), rtL180M (12.5%), rtS202G (3.2%), and rtM204I/V (7%) substitutions, and other resistance mutations were present at low levels (<1%). The frequencies of the resistantance-associated variants show minor fluctuations after the addition of LdT one year after beginning treatment. The frequency of the rtA181T substitution increased to 75.41% in the first year with the addition of LdT, then declined to 28.07% in the second year. In contrast, the rtQ215R substitution increased to 10.89% in the second with multi-drugs. Some certain non-resistance mutations of rtD134N (ranging from 91.97% to 0.21%), rtL145M (ranging from 78.58% to 0.27%) and rtF151Y (ranging from 74.89% to 0.27%) had decreased significantly with the addition of LdT. However, the frequency of the rtS223A mutation increased significantly (ranging from 18.14% to 77.62%) with the addition of LdT (Figure 2B).

**Figure 3:**
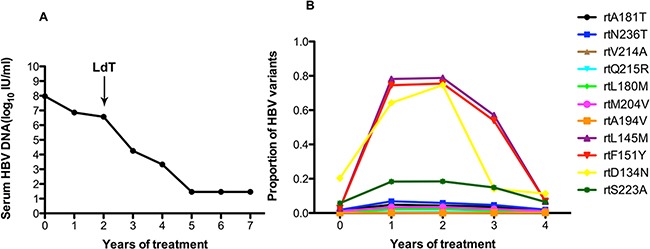
The dynamic changes in HBV DNA levels and resistance-associated variants in the RT domains during ADV+LdT treatment in patient 3 **A.** The serum HBV DNA level declined gradually and was undetectable after 5 years of treatment. **B.** This patient added LdT at the end of the 2 years. At baseline, this patient harbored the rtA181T (1.4%), rtN236T (1.8%), rtS202G (1%), and rtM204I/V (1%) substitutions, and other resistance mutations were present at low levels (<1%). The frequencies of the rtA181T and rtN236T mutations increased during ADV monotherapy and declined when LdT was added, but the fluctuations were minor. Some non-resistance-associated mutations of rtD134N (ranging from 20.33% to 74.63%), rtL145M (ranging from 2.83% to 78.82%), rtF151Y (ranging from 2.92% to 75.51%) and rtS223A (ranging from 5.77% to 18.44%) increased significantly with ADV monotherapy, then declined with the addition of LdT.

Patient 3 responded suboptimally to ADV: the serum DNA level declined by less than 2 log10 IU/ml with 2 years of ADV monotherapy. Following the addition of LdT at the end of the 2 years, the serum DNA level declined gradually and was undetectable after 5 years (Figure 3A). Figure 3B shows that this patient harbored the rtA181T (1.4%), rtN236T (1.8%), rtS202G (1%), and rtM204I/V (1%) substitutions at baseline, and other resistance mutations were present at low levels (<1%). The frequencies of the rtA181T and rtN236T mutation increased during ADV monotherapy and declined when LdT was added, but the fluctuations were minor. Some non-resistance-associated mutations of rtD134N (ranging from 20.33% to 74.63%), rtL145M (ranging from 2.83% to 78.82%), rtF151Y (ranging from 2.92% to 75.51%)and rtS223A (ranging from 5.77% to 18.44%) increased significantly with ADV monotherapy, then declined with the addition of LdT.

**Figure 4:**
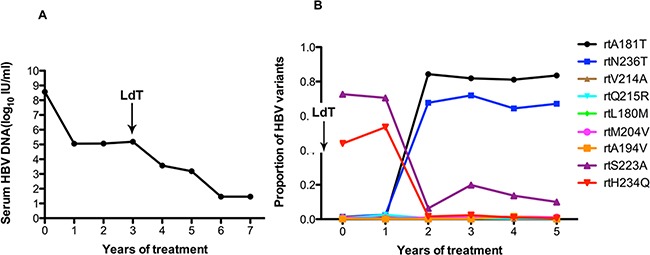
The dynamic changes in HBV DNA levels and resistance-associated variants in the RT domains during ADV+LdT treatment in patient 4 **A.** The serum HBV DNA level was undetectable after 6 years of treatment. **B.** This patient added LdT at the end of the second year. At baseline, patient 4 harbored the rtA181T (1.2%), rtN236T (1.4%), and rtV214A (1.2%) substitutions, and other resistance mutations were present at low levels (<1%). The frequencies of the rtA181V and rtN236T mutations in the HBV RT region were significantly elevated (up to 84.35% and 67.67%, respectively) after two years of ADV monotherapy and did not decreased after the addition of LdT. The frequencies of the rtL180M and rtM204V mutations fluctuated slightly in combination with LdT.

As in patient 3, the serum HBV DNA of patient 4 had declined to 10^5^ IU/ml at the end of one year of ADV treatment but remained unchanged at the end of the second year. After the addition of LdT at the end of the second year, the serum DNA level declined gradually and became undetectable after 6 years of treatment (Figure 4A). At baseline, patient 4 harbored the rtA181T (1.2%), rtN236T (1.4%), and rtV214A (1.2%) substitutions, and other resistance mutations were present at low levels (<1%). The frequencies of the rtA181V and rtN236T mutations in the HBV RT region were significantly elevated (up to 84.35% and 67.67%, respectively) after two years of ADV monotherapy, and did not decrease after the addition of LdT. The frequencies of the rtL180M and rtM204V mutation fluctuated slightly after the addition of LdT (Figure 4B).

**Figure 5:**
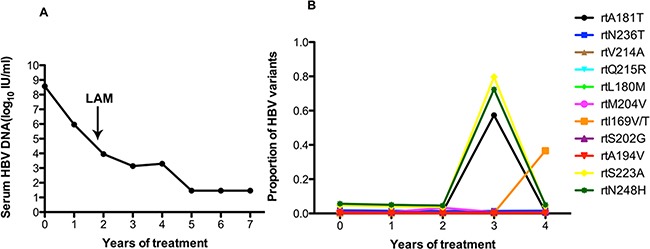
The dynamic changes in HBV DNA levels and resistance-associated variants in the RT domains during ADV+LAM treatment in patient 5 **A.** The serum DNA level declined gradually and was undetectable after 5 years of treatment with the addition of LdT after 1 years and 9 months. **B.** This patient harbored the rtA181T (1.2%), rtN236T (1.8%), rtS202G (1.1%), and rtM204I/V (1.1%) substitutions, and other resistance mutations were present at low levels (<1%) at baseline. The frequency of the rtA181V mutation significantly fluctuated (ranging from 57.34% to 1.14%) after combination with LdT. This phenomenon usually appeared in non-resistance mutations of rtS223A (ranging from 79.67% to 4.41%) and rtN248H (ranging from 72.38% to 5.03%).

Figure 5A shows that the HBV DNA had declined to 10^5^ IU/ml after one year, but viral replication persisted during ADV monotherapy. The serum DNA level declined gradually and was undetectable after 5 years of treatment with the addition of LdT after 1 years and 9 months. The results in Figure 5B demonstrated that patient 5 harbored the rtA181T (1.2%), rtN236T (1.8%), rtS202G (1.1%), and rtM204I/V (1.1%) substitutions, and other resistance mutations were present at low levels (<1%) at baseline. The frequency of the rtA181V mutation significantly fluctuated (ranging from 57.34% to 1.14%) after combination with LdT. This phenomenon usually appeared in non-resistance mutations of rtS223A (ranging from 79.67% to 4.41%) and rtN248H (ranging from 72.38% to 5.03%) (Figure 5B).

**Figure 6:**
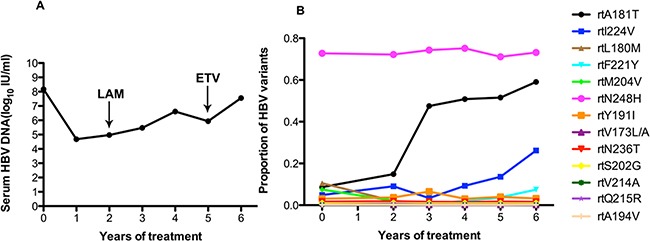
The dynamic changes in HBV DNA levels and resistance-associated variants in the RT domains during ADV+LAM treatment in patient 6 **A.** The serum DNA level declined to 10^4^ log10 IU/ml with 2 years of ADV monotherapy. Then, after combination with LAM at the end of the 2 years, the serum DNA level increased to 10^6^ IU/ml. Thus, in this patient treated with ETV and ADV, the HBV DNA level increased to 10^7^ IU/ml after 1 year. **B.** At baseline, this patient harbored the rtA181T (8.7%), rtN236T (1.7%), rtL180M(10.5%), rtM204I/V (7.5%), and rtV173A/M (1.6%) substitutions, and other resistance mutations were present at low levels (<1%). The frequency of the rtA181T mutation significantly increased after the second year, with a 50% increase during follow-up treatment. The frequency of the rtI224V mutation increased slowly. The frequency of the rtN248H mutation was high (70%). The fluctuations in other resistance mutations were relatively minor.

Patient 6 responded to ADV: the serum DNA level declined to 10^4^ log10 IU/ml with 2 year of ADV monotherapy. Then, after combination with LAM at the end of the 2 years, the serum DNA level increased to 10^6^ IU/ml. Thus, this patient was treated with ETV and ADV, the HBV DNA level increased to 10^7^IU/ml after 1 year (Figure 6A). Figure 6B showed that this patient harbored the rtA181T (8.7%), rtN236T (1.7%), rtL180M(10.5%), rtM204I/V (7.5%), and rtV173A/M (1.6%) substitutions at baseline, and other resistance mutations were present at low levels (<1%). The frequency of the rtA181T mutation significantly increased after the second year, with a 50% increase during follow-up treatment. The frequency of the rtI224V mutation increased slowly. The frequency of the rtN248H mutation was high (70%). The fluctuations in other related resistance mutations were relatively minor.

**Figure 7:**
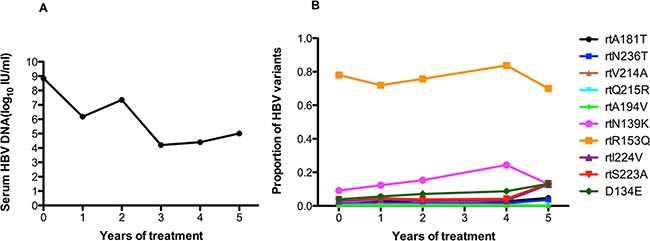
The dynamic changes in HBV DNA levels and resistance-associated variants in the RT domains during ADV monotherapy in patient 7 **A.** The serum HBV DNA level fluctuated around 10^4^ to 10^5^ IU/ml during long-term ADV monotherapy. **B.** At baseline, this patient harbored the rtA181T (1.2%), rtN236T (1.5%), and rtV214A (1%) substitutions, and other resistance mutations were present at low levels (<1%). The frequencies of the rtA181V and rtN236T mutations increased slowly (ranging from 1.22% to 4.78% and 1.51% to 3.57%) after long-term treatment. The rtR153Q mutation was always present at a high frequency (70%). The frequency of the rtN139K mutation slowly increased after one year of treatment. The frequencies of the rtI224V, rtS223A, rtD134E mutations slowly increased after 4 years of treatment. The fluctuations in the frequency of other resistance mutations were relatively minor.

The serum HBV DNA level in patient 7 fluctuated around 10^4^ to 10^5^ IU/ml during long-term ADV monotherapy (Figure 7A). At baseline, this patient harbored the rtA181T (1.2%), rtN236T (1.5%), and rtV214A (1%) substitutions, and other resistance mutations were present at low levels (<1%). The frequencies of the rtA181V and rtN236T mutations increased slowly (ranging from 1.22% to 4.78% and 1.51% to 3.57%) after long-term treatment. The rtR153Q mutation was always present at a high frequency (70%). The frequency of the rtN139K mutation slowly increased after one year of treatment. The frequencies of the rtI224V, rtS223A, rtD134E mutations slowly increased after 4 year of treatment. The fluctuations in the frequencies of other related resistance mutations were relatively minor (Figure 7B).

**Figure 8:**
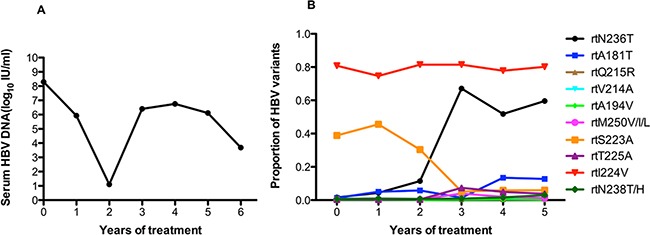
The dynamic changes in HBV DNA levels and resistance-associated variants in the RT domains during ADV monotherapy in patient 8 **A.** The patient underwent long-term ADV monotherapy, then experienced virological breakthrough after 3 years of treatment. The viral load remained at 10^6^ IU/ml in follow-up therapy. **B.** This patient harbored the rtA181T (1.3%) and rtN236T (1.6%) substitutions at baseline, and other resistance mutations were present at low levels (<1%). It is noteworthy that the frequency of the rtN236T mutation began to increase significantly after two years of ADV monotherapy and was present in up to 50% of circulating viruses during follow-up treatment. The frequency of the rtA181T mutation was more than 10% after 4 years of treatment. The frequency of the rtI224V mutation was always high (70~80%).

As in patient 7, patient 8 underwent long-term ADV monotherapy, then experienced a virological breakthrough after 3 years of treatment. The viral load remained at 10^6^ IU/ml in follow-up therapy (Figure 8A). As shown in Figure 8B, this patient harbored the rtA181T (1.3%) and rtN236T (1.6%) substitutions at baseline, and other resistance mutations were present at low levels (<1%). It is noteworthy that the frequency of the rtN236T mutation began to increase significantly after two years of ADV monotherapy and was present in up to 50% of circulating viruses during follow-up treatment. The frequency of the rtA181T mutation was more than 10% after four years of treatment. The frequency of the rtI224V mutation was always high (70-80%) (Figure 8B).

## DISCUSSION

The aim of treatment is to inhibit viral replication and ultimately achieve HBsAg seroclearance. However, with the long-term use of antiviral drugs, resistance has become a major barrier to successful treatment and thus promotes disease progression [12]. The patients who underwent ETV monotherapy have been described comprehensively [13]. The ADV group included patients for whom multidrug therapy had previously been unsuccessful. The frequency of mutations was higher under monotherapy compared with under multidrug therapy [14]. There are some studies on ADV-resistance mutations [11], but there are few studies about HBV RT region resistantance-assocaited mutations in multi-drugs combinations.

According to the results of the high-sensitivity UDPS analysis, two resistance-associated variants (rtA181V/T, rtN236T) were detected at low prevalence in all treatment-naïve patients (ranging from 1.1% to 6.1%). Some studies about pre-existing mutations have been published [15, 16]. In one article, the most commonly detected mutations were M204V/I(9/14) and M250V/I(11/14); other documented mutations include A181T/V(7/14) and N236T(3/14) [17]. These results are similar to those of Nishijima N et al. in that some low-abundance mutations were present at baseline, although we report different variant sites. Whether the frequencies of these mutations are associated with populations or genotypes remains to be further verified. Only limited information is currently available regarding pre-existing mutations ifentified by UDPS.

The patients who responded poorly to ADV were given supplemental treatment with LAM, LdT or ETV to inhibit viral replication. In this long-term follow-up study, we found that the dynamics of the quasispecies variation in the HBV RT region are heterogeneous and patient-specific. This study included four patients on ADV+LdT therapy, two patients on ADV+LAM and two patients on ADV monotherapy. Six patients who were on multidrug therapies cleared virus after 3 or 4 years of treatment. Four patients (NOs.1, 2, 3, 4) on ADV and LdT ultimately cleared the virus. The virus communities in patients 1 and 2 harbored the A181T/V(4.7%/4.7%), N236T(6%/5.7%), L180M(2.4%/12.5%) and M204I/V(3.4%/7%), while those in patients 3 and 4 harbored only the A181T/V(1.4%/1.2%), and N236T(1.8%/1.4%). During ADV monotherapy, these substitutions increased in frequency. When LdT was added to the regimen, the frequencies of these substitutions declined (patient 1-3), except in patient 4. This result suggests that the fitness gain associated with drug resistance cannot be reversed, and these patients ultimately experienced viral breakthrough. Patients 5 and 6 were treated with ADV and LAM to suppress viral replication. They harbor the A181T/V(1.2%/8.7%) and N236T(1.8%/1.7%) substitutions; that of patient 6 harbored the L180M(10.5%) and M204I/V(7.5%), which are associated with resistance to LAM [18]. Patient 6 experienced viral breakthrough while on LAM and ETV. Patients 7 and 8 remained on ADV monotherapy for personal reasons.

During treatment, the frequency of the rtA181T resistance sites increased in six of the eight patients (NOs. 1-6). Two patients (NOs. 4 and 8) in whom the rate of rtA181T resistance was increasing showed high levels of rtN236T. One patient (NO. 8) experienced virological breakthrough. This research demonstrates that the rtA181T mutation can cause drug resistance and increase viral replication, which are mainly dependent on the mutations in the overlapping surface gene [19]. In addition to the common substitutions, some unknown amino acid substitutions, such as rtD134N, rtL145M/S, rtF151Y/L, rtR153Q, rtS223A, rtI224V, and rtN248H, need to be further verified. In addition to viral quasispecies composition, many factors can affect treatment outcome, such as host immune status, baseline HBV DNA level and genotype [20–22].

For long-term therapy, which is associated with the emergence of multidrug-resistant HBV mutations, tenofovir disoproxil fumarate (TDF) is effective as an acyclic nucleotide analogue in clinical practice [23, 24]. After a long-term follow-up study, we did not detect the emergency of TDF-resistance-associated variants (rtA194T, rtP177G, and rtF249A) [7, 8], suggesting that this study will provide the basis for changing to a different medication in clinical trials. The research also revealed bone loss caused by proximal tubular dysfunction during TDF therapy [25, 26], so longitudinal follow-up studies are needed.

In conclusion, the dynamics of the multidrug-resistance-related variation in the RT region in HBV quasispecies are heterogeneous and complex. Hepatitis B multidrug therapy will eventually result in virological suppression; on the other hand, resistantance will arise in patients with long-term HBV DNA positive. Thus, it is urgent that the potential for resistance be considered when deciding whether to add more antiviral drugs to a regimen or transition to more potent drugs.

## MATERIALS AND METHODS

### Ethics statement

The Medical Ethics Committee of Peking University First Hospital approved the study, and all the participants in this study written informed consent. The study was in compliance with the Helsinki Declaration.

### Study patients

One hundred and seven patients treated with NAs were recruited from the Department of Infectious Disease of Peking University First Hospital (China), including 61 patients treated with ETV and 46 patients treated with ADV. The former patients regularly received ETV (0.5 mg, qd) for at least 7 years; the latter patients received ADV at a dose of 10 mg daily, alone or combined with lamivudine, telbivudine or entecavir in the case of ADV treatment failure. The major inclusion criteria were no previous NA treatment and NA therapy for 7 years without interruption. The exclusion criteria were serious liver-related complications (decompensated liver cirrhosis and hepatocellular carcinoma), co-infection with human immunodeficiency virus (HIV) or hepatitis C virus (HCV), and a lack of comprehensive samples at testing time points. The clinical definition was as follows: complete virological response (CVR) was defined as undetectable HBV DNA levels after 24 weeks of treatment, whereas the partial virological response (PVR) was defined as being HBV DNA positive after 24 weeks with a viral load that is lower by >2 log10 IU/ml compared to the baseline. Virological breakthrough was defined as an increase in the HBV DNA levels by more than 1 log10 IU/ml compared to the nadir level while on therapy.

The ETV group included a total of 32 treatment-naïve patients, as shown in the published article. The ADV group included a total of 14 patients. Additionally, 61 serum samples from naïve and NAs-experienced individuals were assayed by UDPS: 14 samples were taken from all patients at baseline, and 47 were taken after the sequential inter-treatment of PVRs.

### Virological indicators

Indicators were tested at baseline and at selected time points (0.5, 1, 2, 3, 4, 5, 6 and 7 years) after treatment. Alanine aminotransferase and aspartate transaminase were quantified on an automatic biochemical analyzer. HBV DNA was quantified by a Cobas TaqMan assay (Roche Diagnostics, Basel, Switzerland), and the detection limit was 20 IU/mL. Other serological markers (HBsAg, anti-HBsAg, HBeAg, anti-HBe, anti-HBc) were measured using ELISA kits (Abbott Laboratories, Chicago, IL, USA). HBV genotypes were determined using NCBI data according to comparing the generated preS/S gene sequences.

### PCR amplification and UDPS data

HBV DNA was extracted from 1 mL serum samples according to the manufacturer's instructions (QIAamp UltraSens Virus kit, Qiagen, Germany). The first PCR fragment (nt84 to 997) was amplified with the primers HBVRTfw1: 5'-GGCTCCAGTTCAGGAACAGT-3' and HBVRTrv1: 5'-GCAAAGCCCAAAAGACCCACAAT-3'. The second PCR fragment (nt 515 to 898) was amplified with the primers HBVRTfw2: 5'-CTACCAGCACGGGACCAT-3' and HBVRTrv2: 5'-TCCTGTGGTAAAGTACCCCA-3'. The following cycling profile was used for PCR: 40 cycles of 98°C for 20 seconds, and 60°C for 30 seconds, with a final extension of 72°C for 5 minutes. The products were analyzed via electrophoresis through 1% agarose gel and ethidium bromide staining. Then, the PCR amplicons were purified with the QIAquick PCR purification kit (Qiagen) and quantified with an Agilent 2100 bioanalyzer (Agilent Life Science, Santa Clara, CA). Afterward, the amplicons were sequenced on the MiSeq platform (Illumina, American).

Because of the high rates of sequencing error and homopolymeric bias, all UDPS reads were subjected to additional error correction by the Sanger method (Applied Biosystems, Foster City, CA). All reads from the UDPS and Sanger methods were aligned to homologous sequences from GenBank. In UDPS, every sample was given a barcode tag to distinguish between samples from different patients. The paired-end (PE) reads were combined by FLASH (v1.2.7) and subjected to quality control using FastQC. The combined reads that included more than 20% low-quality bases (quality score < 20) or has more than 5 extra-low-quality bases (quality score less than 5) were discarded. The obtained libraries varied from 480256 to 155902 reads, with a mean value of 78410 reads per sample. Reads that aligned to reference sequences with >=90% coverage were reserved for further analysis. Only sequences that passed the quality filters were used to calculate coverage.

## Abbreviations

HBV: Hepatitis B virus
CHB: Chronic Hepatitis B
NA: nucleos(t)ide analogue
TDF: Tenofovir Disoproxil Fumarate
ADV: adefovir
HIV: human immunodeficiency virus
HCV: hepatitis C virus
CVR: completed virological response
PVR: partial virological response
UDPS: ultra-deep pyrosequencing
